# Effects of Real-Time (Sonification) and Rhythmic Auditory Stimuli on Recovering Arm Function Post Stroke: A Systematic Review and Meta-Analysis

**DOI:** 10.3389/fneur.2018.00488

**Published:** 2018-07-13

**Authors:** Shashank Ghai

**Affiliations:** Institute for Sports Science, Leibniz University Hannover, Hannover, Germany

**Keywords:** cueing, stability, rehabilitation, cognitive-motor interference, hemiplegia, spasticity, paresis

## Abstract

**Background:** External auditory stimuli have been widely used for recovering arm function post-stroke. Rhythmic and real-time auditory stimuli have been reported to enhance motor recovery by facilitating perceptuomotor representation, cross-modal processing, and neural plasticity. However, a consensus as to their influence for recovering arm function post-stroke is still warranted because of high variability noted in research methods.

**Objective:** A systematic review and meta-analysis was carried out to analyze the effects of rhythmic and real-time auditory stimuli on arm recovery post stroke.

**Method:** Systematic identification of published literature was performed according to PRISMA guidelines, from inception until December 2017, on online databases: Web of science, PEDro, EBSCO, MEDLINE, Cochrane, EMBASE, and PROQUEST. Studies were critically appraised using PEDro scale.

**Results:** Of 1,889 records, 23 studies which involved 585 (226 females/359 males) patients met our inclusion criteria. The meta-analysis revealed beneficial effects of training with both types of auditory inputs for Fugl-Meyer assessment (Hedge's g: 0.79), Stroke impact scale (0.95), elbow range of motion (0.37), and reduction in wolf motor function time test (−0.55). Upon further comparison, a beneficial effect of real-time auditory feedback was found over rhythmic auditory cueing for Fugl-meyer assessment (1.3 as compared to 0.6). Moreover, the findings suggest a training dosage of 30 min to 1 h for at least 3–5 sessions per week with either of the auditory stimuli.

**Conclusion:** This review suggests the application of external auditory stimuli for recovering arm functioning post-stroke.

## Introduction

According to World health organization, stroke accounts as the third main cause of disability across the world (1). The incidence of stroke related disability have almost doubled in the developing countries in the past decade (2, 3). The disability affects basic day to day life activities (4), which further increase dependency (5), anxiety, depression (6), social isolation (7), and promote a poor quality of life (8, 9). Moreover, the disability inflicts substantial economic burden on patients (10).

Typically, patients affected from stroke exhibit sensorimotor dysfunctions on the contralateral side of the affected brain region (11). These deficits can be exhibited focally, segmentally, unilaterally, or bilaterally (12). The symptoms are typically characterized by progressive inefficient movement synergy patterns (13), abnormal muscle tone (14), force production (15), compromised dexterity (16), poor coordination (17), and more (18). Moreover, hyper/hypokinetic movement disorders are also common [see Handley et al.,(12)]. Additionally, cognitive and sensory dysfunctions are also common in patients with stroke (19). Despite advancements in rehabilitation, poor prognosis in stroke is still prevalent, especially for recovering arm function (5, 20). Studies suggest that upper limb recovery is an important predictor for determining the health status outcome, and quality of life for stroke patients (21, 22).

The poor gross and fine motor performance in upper extremities can be due to abnormal co-contraction of antagonists/agonists (23), disruptions in force production/adaptation (24), and regulation of stretch reflex (15, 25). Besides, these musculoskeletal dysfunctions can considerably impair joint kinematics (26, 27). According to Hara et al. (28) impaired activation of motor units in terms of firing rate and synchronization might result in such deficits. Furthermore, as the disease progresses, these changes increase fatigue (29), reduce coordination (30), and with the progression of time promote development of joint contractures (31), and subluxations/dislocations (32). Likewise, discrepancies in sensory perceptions, memory, cognition, and behavior further impact the prognostic outcome of a stroke patient (33–35).

Neuroimaging studies suggest site specific lesions and silent infarcts at medial temporal lobe (36), gray (37), and white matter (38), further leading to a wide array of cognitive dysfunctions (39) [see Makin, (40) and Sperber and Karnath (41).] Similarly, deficits in corticospinal (42, 43), thalamocortical (44), superior occipito-frontal (41), and superior-longitudinal pathways (45), might overload the already impaired cognitive-motor pathways. Such a constraining impact on the impaired cognitive pathways might increase “internal” conscious monitoring by the patients to control their movements [see movement re-investment 46–48)]. This increase in attention is aimed to safeguard the stability of a movement (49, 50), it retrospectively impairs autonomic execution of a movement and promotes movement failure (46–48). Likewise, dysfunctions in sensory perception could affect perceptuomotor representations in the brain, thereby affecting motor planning and execution (35). Together, these cognitive and sensorimotor dysfunctions affect the prognosis of a stroke patient.

Common treatment strategies to curb cognitive motor dysfunctions in stroke patients include training with virtual-reality (51), mental imagery (52), biofeedback (53), physical therapy (54), exercise (55), prosthesis (56–58), dual-task priority training, and more (59). Recently studies have tried to enhance the stroke recovery by simultaneously addressing the sensory deficits with motor rehabilitation by applying external sensory stimulation as a neuro-prosthetic (59–62). Studies have analyzed the effects of different sensory stimuli in auditory, visual and tactile domain on motor performance (59, 61, 62). However, the literature predominantly supports the beneficial role of auditory stimuli (50, 63, 64). The main reasons which underlie the beneficial effects are thought to be multifaceted. Firstly, rich neuroanatomical interconnectivity has been reported between auditory and motor cortex (65–67). Here, inference can be drawn from literature evaluating auditory startle reflex on animal models (68, 69). Studies using Double-labeling experiments have revealed that cochlear root neurons in the auditory nerve can project bilaterally to sensorimotor paths, including synapsing on reticulospinal neurons (65, 68, 70). Likewise, patterns of thalamocortical and corticocortical inputs unique to auditory cortex have also been reported [for a detailed review see (71)]. In humans, neuroimaging data confirms the presence of cortico-subcortical network involving putamen, supplementary motor area, premotor cortex, and the auditory cortex especially for perceiving and processing rhythmic auditory stimuli (72–75). Secondly, the human auditory system can consistently perceive auditory cues 20–50 ms faster as compared to its visual and tactile counterparts (76–78). Thirdly, the auditory system has a strong bias to identify temporal patterns of periodicity and structure as compared to other sensory perceptual systems (78–80). For instance, auditory rhythmic perception has been reported to exist well beyond the limits of temporal resolution of visual modalities i.e., when periodicities are presented at a rate of ~300–900 ms (80, 81).

In the literature, however, rhythmic auditory cueing (67), and real-time kinematic auditory feedback (82), also termed as sonification, are the most widely studied approaches in upper limb stroke rehabilitation. Both the methods possess differential influence over neurophysiological and musculoskeletal domains. Firstly, rhythmic auditory cueing can be defined as repetitive isosynchronous stimulations applied with an aim to simultaneously synchronize motor execution (83, 84). Here, neuroimaging data for rhythmic auditory stimuli suggests facilitated activations in premotor cortex, insula, cuneus, supplementary motor area, cerebellum, and basal ganglia (73, 80, 85–87). Moreover, training with rhythmic auditory cueing has been reported to modulate neuromagnetic β oscillations (88, 89), biological motion perception (82, 90), auditory-motor imagery (91–93), shape variability in musculoskeletal activation patterns (94), cortical reorganization, neural-plasticity (95, 96), and also movement specific re-investment (97). Real-time kinematic auditory feedback on the other hand is a comparatively new approach. Such type of an intervention involves mapping of movement parameters on to the sound components, such as pitch, amplitude with a very minimal or no latency (82). The feedback has been reported to alleviate sensory perceptions like proprioception (98), by enhancing sensorimotor representation while facilitating activations in action observation system (90), and inducing neural plasticity (99). Moreover, the feedback has been reported by Effenberg et al. (82) to extend the benefits of discrete rhythmic auditory cueing stimuli. Here, the authors suggest that the continuous flow of information might allow a participant to better perceive their movement amplitudes and positioning, thereby resulting in development of both feedback and feed-forward models (82). Moreover, by allowing additional influence over the action observation system the real-time auditory stimuli might also enrich the internal stimulation of the executed movement (50, 82, 90). This methodology involves delivering action relevant auditory feedback, where the characteristics of stimuli (e.g., frequency, amplitude) are mapped to the specific joint kinematics in real-time, for an example see (98). Schmitz et al. (90) in a neuroimaging study reported that observation of a convergent audio (sonification)-visual feedback led to enhanced activations in fronto-parietal networks, action observation system i.e., superior temporal sulcus, Broadman area 44, 6, insula, precentral gyrus, cerebellum, thalamus and basal ganglia (90). The authors mentioned that the multimodal nature of the stimuli can enhance the activation in areas associated with biological motion perception and in sub-cortical structures involving striatal-thalamic frontal motor loop. This then might improve perceptual analysis of a movement thereby resulting in efficient motor planning and execution (90).

Till date, no study has analyzed the influence of real-time auditory feedback on upper limb recovery post-stroke. Moreover, no study has compared the influence of rhythmic and real-time auditory stimuli on upper limb recovery post stroke. This information might serve to be an important source of information for future research and for developing efficient rehabilitation protocols in stroke community. Only four systematic reviews have analyzed the influence of rhythmic auditory stimulations on arm recovery post stroke (100, 101–103), in which only two reviews included a statistical meta-analysis (102, 103). In these studies limitations persisted in terms of meta-analysis approach i.e., no heterogeneity analysis. Therefore, interpretation of results from the statistical analyses might indicate biasing. Therefore, the aim of the present systematic review and meta-analysis is to develop a state of knowledge where both qualitative and quantitative data for different auditory stimuli delivery methods can be interpreted for the use of stroke patients and medical practitioners alike. Moreover, a meta-analysis approach will be used to determine specific training dosage for auditory stimuli in recovering arm function post-stroke.

## Methods

This systematic review and meta-analysis was conducted according to the guidelines outlined by PRISMA statement: Preferred Reporting Items for Systematic Reviews and Meta-analysis (104).

### Data sources and search strategy

Academic databases: Web of science, PEDro, EBSCO, MEDLINE, Cochrane central register of controlled trials, EMBASE, and PROQUEST were searched from inception until December 2017. A sample search PICOS strategy for the review has been provided in (Table 1) (105).

**Table 1:** Sample search strategy EMBASE.

**PICOS**	**DATABSE**	**EMBASE**
	**DATE**	**10/12/2017**
	**STRATEGY**	**#1 AND #2 AND #3 AND #4 AND #5 AND #6**
P	#1	(“Stroke” OR “Apoplexy” OR “CVA” OR “Cerebral Stroke” OR “Cerebrovascular accident” OR “Cerebrovascular Accident, Acute” OR “Cerebrovascular Apoplexy” OR “Cerebrovascular Stroke” OR “Stroke, Acute” OR “Vascular Accident, Brain” OR “Hemiplegia, Crossed” OR “Hemiplegia, Flaccid” OR “Hemiplegia, Spastic” OR “Hemiplegia, Transient” OR “Monoplegia” OR “Upper Extremity Paresis” OR “Muscular Paresis” OR “Muscle Paresis” OR “Monoparesis” OR “Hemiparesis”)/de OR (Stroke OR Apoplexy OR CVA OR Cerebral Stroke OR Cerebrovascular accident OR Cerebrovascular Accident, Acute OR Cerebrovascular Apoplexy OR Cerebrovascular Stroke OR Stroke, Acute OR Vascular Accident, Brain OR Hemiplegia, Crossed OR Hemiplegia, Flaccid OR Hemiplegia, Spastic OR Hemiplegia, Transient OR Monoplegia OR Upper Extremity Paresis OR Muscular Paresis OR Muscle Paresis OR Monoparesis OR Hemiparesis):ti,ab
I	#2	(“rhythmic auditory cueing” OR “rhythmic auditory cueing” OR “rhythmic acoustic cueing” OR “rhythmic auditory entrainment” OR “metronome cueing” OR “metronome” OR “rhythmic metronome cueing” OR “acoustic stimulus” OR “acoustic cueing” OR “acoustic cueing” OR “external stimuli” OR “external cueing” OR “external cueing” OR “music therapy” OR “Neurological music therapy” OR “tempo” OR “beat” OR “rhythm” OR “RAC” OR “NMT” OR “real-time auditory feedback” OR “sonification”)/de OR (rhythmic auditory cueing OR rhythmic auditory cueing OR rhythmic acoustic cueing OR rhythmic auditory entrainment OR metronome cueing OR metronome OR rhythmic metronome cueing OR acoustic stimulus OR acoustic cueing OR acoustic cueing OR external stimuli OR external cueing OR external cueing OR music therapy OR Neurological music therapy OR tempo OR beat OR rhythm OR RAC OR NMT OR real-time auditory feedback OR sonification)ti,ab
C	n/a	n/a
O	#3	(“Range of Motion” OR “Passive Range of Motion” OR “Joint Range of Motion” OR “Joint Flexibility” “elbow” OR “shoulder” OR “wrist” OR “Fugl Meyer Assessment” OR “Fugl-Meyer assessment for upper extremity” OR “FMA” OR “Wolf motor assessment” OR “WMA” OR “Wolf motor test” OR “Nine hole peg test” OR “NHPT” OR “9HPT” OR “Action reach arm test” OR “ARAT” OR “Stroke index scale” OR “SIS” OR “BATRAC” OR “Bilateral arm training with rhythmic auditory cueing” OR “Unilateral arm training with rhythmic auditory cueing” OR “Arm reach training” OR “BBT” OR “Box and block test” OR “Motor activity log” OR “MAL” OR “Cincinnati Stroke Scale” OR “Los Angeles Prehospital Stroke Scale” OR “ABCD Score” OR “Canadian Neurological Scale” OR “European Stroke Scale” OR “Hemispheric Stroke Scale” OR “NIH Stroke Scale” OR “Modified Rankin Scale” OR “Stroke Specific Quality of Life Measure” OR “Health Survey SF-36” OR “Health Survey SF-12”)/de OR (Range of Motion OR Passive Range of Motion OR Joint Range of Motion OR Joint Flexibility elbow OR shoulder OR wrist OR Fugl Meyer Assessment OR Fugl-Meyer assessment for upper extremity OR FMA OR Wolf motor assessment OR WMA OR Wolf motor test OR Nine hole peg test OR NHPT OR 9HPT OR Action reach arm test OR ARAT OR Stroke index scale OR SIS OR BATRAC OR Bilateral arm training with rhythmic auditory cueing OR Unilateral arm training with rhythmic auditory cueing OR Arm reach training OR BBT OR Box and block test OR Motor activity log OR MAL OR Cincinnati Stroke Scale OR Los Angeles Prehospital Stroke Scale OR ABCD Score OR Canadian Neurological Scale OR European Stroke Scale OR Hemispheric Stroke Scale OR NIH Stroke Scale OR Modified Rankin Scale OR Stroke Specific Quality of Life Measure OR Health Survey SF-36 OR Health Survey SF-12);ti,ab
S	#6	(“intervention study” OR “cohort analysis” OR “longitudinal study” OR “cluster analysis” OR “crossover trial” OR “cluster analysis” OR “randomized trial” OR “major clinical study”)/de OR (longitudinal OR cohort OR crossover trial OR cluster analysis OR randomized trial OR clinical trial OR controlled trial);ti,ab
	#4	(“rehabilitation” OR “treatment” OR “rehab” OR “management” OR “therapy” OR “physiotherapy” OR “physical therapy” OR “prevention” OR “risk prevention”)/de OR (rehabilitation OR treatment OR rehab OR management OR therapy OR physiotherapy OR physical therapy OR prevention OR risk prevention);ti,ab
	#5	(“age groups” OR “adolescent” OR “young” OR “elderly” OR “old” AND (“gender” OR “male” OR “female”)/de OR [age groups OR adolescent OR young OR elderly OR old AND (gender OR male OR female)];ti;ab

### Data extraction

Upon selection for review, the following data were extracted from each article; author, date of publication, selection criteria, sample size, sample description (gender, age, health status, duration of stroke), applied intervention, characteristics of auditory stimuli i.e., rhythmic/real-time, applied dual-task (if any), outcome measures, results, and conclusions. The data were then summarized and tabulated (Table 2).

**Table 2:** Effects of auditory stimuli on arm function post-stroke.

**Author**	**Research question(s)/ hypothesis**	**Sample description, age: (M ± S.D)**	**PEDro**	**Disease duration**	**Assessment tools**	**Research design**	**Auditory stimuli characteristics**	**Conclusion**
Bang (106)	Effect of R-af on arm function in patients affected from stroke	Exp: 4F, 6M (61.3 ± 4.8) Ct: 5F, 5M (58.2 ± 5.1)	9	Exp: 8.9 ± 3.1 years Ct: 10.3 ± 3.7 years	ARAT, FMA, motor activity log (quality of movement, amount of use) and modified Ashworth scale	Pre-test, modified constraint induced movement therapy with/without R-af for 1 h/day, 5 days/week for 4 weeks, post-test	R-af (proportional to reduced pressure by shoulder on the sensor) Frequency faded off with progression from every 1/3rd trial	Significant enhancement in ARAT, FMA, motor activity log (quality of movement, amount of use), modified ashworth scale after training with R-af and in Exp as compared to Ct
Scholz et al. (107)	Effects of R-af on gross motor functions on participants affected from stroke (right hemiparesis).	Exp: 1F (59), 1M (85) Ct: 2M (61.5 ± 3.5)	4	–	FMA, ARAT, BBT, 9-HPT, and SIS	Patients moved their arms in a 3D R-af space, for 9 days of training with R-af (30 min/day)	X axis: Brightness mapped, increased from left to right Y axis: Pitch mapped, increased from bottom to up Z axis: Volume, increased when closed to the participant	Exp: Enhancements were observed for participants in FMA, ARAT, 9-HPT, and SIS Ct: No enhancements were observed for, but minimally for one participants in FMA, and the other in SIS
Malcolm et al. (108)	Effect of RAC on arm kinematics in patients affected from stroke	5M (72.8 ± 6.5)	4	0.7 ± 0.4 years	Movement time, reach velocity, wolf motor function test, FMA, and motor activity log	Pre-test, 1-h session followed by 2 h of home training, 3 times/week for 2 weeks with RAC and reaching performed in sagittal, frontal and diagonal planes, post-test	RAC at patients preferred pace of movement	Significantly enhanced reaching velocity, FMA, and motor activity log after training with RAC Significantly reduced reaching time, wolf motor function test performance time after training from RAC
Speth (109)	Effect of RAC on arm reaching in patients affected from stroke	8 stroke patients	4	–	BBT for (paretic/non-paretic side)	BBT performance with/without RAC i.e., waltz music, metronome	RAC (200 bpm), waltz music (200 bpm) cueing	Enhanced performance for BBT with waltz music>RAC>no feedback for both paretic and non-paretic arms
	Effect of RAC on robot-assisted arm reaching in patients affected from stroke	11F, 22M (51.6 ± 15.9), severe: 18, moderate: 8, mild: 8 Exp: 14 [A; severe (11), moderate (4), mild (4)] [B: 2–6 months' post stroke (8), >12months post stroke (9)] Ct: 14 [A: severe (6), moderate (4), mild (4)] [B: 2-6 months' post stroke (9), >12months post stroke (5)]		1.2 ± 1.3 years	BBT, 9-HPT, and intrinsic motivation inventory	Pre-test, robot assisted arm training “Amadeo” with (Exp)/without (Ct) RAC (polymetric music, game-related action feedback) for 45 min for 9 times for 3–4 weeks, post-test, retention measurement after 8 weeks' post-test	RAC by polymetric music (rhythmic adaptability to multi-joint movements in hand and finger movement e.g., first 3/4 m containing 2 bars, second 2/4 m containing 3 bars, 3rd 3/8 m containing 4 bars: all sounds played in one absolute time frame) and game related sounds (error feedback, natural sounds) together	Significant enhancement in mean BBT for moderate and mild affected patients, for Exp as compared to Ct Significant reduction in mean box and block test for severely affected patients, for Ct as compared to Exp Enhancement in 9-HPT for Exp as compared to Ct Significant enhancement in intrinsic motivation inventory for (interest/enjoyment, perceived competence, relaxation, perceived choice) for Exp as compared to Ct
Scholz et al. (60)	Effects of R-af on gross motor functions on participants affected from stroke (right hemiparesis).	Exp: 7F, 8M (68.8 ± 13.6) Ct: 4F, 6M (72.2 ± 8.4)	6	Exp: 32.5 days Ct: 28 days	FMA, ARAT, BBT, 9-HPT, and SIS	Patients moved their arms in a 3D sonification space, for 10 days of sonification training (30 min/day).	X axis: Brightness mapped, increased from left to right Y axis: Pitch mapped, increased from bottom to up Z axis: Volume, increased when closed to the participant	Exp: Significant enhancements were observed for participants in movement smoothness, FMA, SIS as compared to Ct Enhancements were observed in ARAT, BBT and 9-HPT Ct: No significant enhancements were observed post sham training
van Delden et al. (110)	Effects of RAC on arm reaching in patients affected from stroke	Exp: 8F, 11M (62.6 ± 9.8) Ct I: 3F, 16M (56.9 ± 12.7) CT II: 8F, 14M (59.8 ± 13.8)	7	Exp: 7.8 ± 4.9 weeks Ct I: 9.2 ± 6.8 weeks Ct II: 11.1 ± 6.8 weeks	ARAT, motricity index, FMA, 9-HPT, Erasmus modification of Nottingham sensory assessment, motor activity log test and SIS	Pre-test, BATRAC (Exp), modified constrained induced movement therapy (Ct I), conventional therapy (Ct II), for 60 min session, 3 times/week, post-test, 6 weeks follow up post-test	RAC (rhythmic flexion-extension at the wrist joint)	Significant enhancement in ARAT with BATRAC No differences in between Exp, Ct I, Ct II
Schmitz et al. (111)	Effect of R-af on reaching task in patients affected from stroke	Exp: 1F, 3M (65 ± 14.8) Ct: 3F (56 ± 5.3)	4	-	ARAT, 9HPT, and BBT	I: Reaching and retraction task by affected arm. II: Patients repositioned a ball on objects of different shapes. Training for 5 days for 5 sessions of 20 min each	Arm velocity: modulates amplitude of sound Elevation angle: modulates frequencies between 133.3 and 266.6 Hz. Radial arm amplitude: modulates brightness	Significant enhancements were observed in BBT for Exp as compared to Ct. Enhancements were observed in 9HPT and ARAT. Ct: No significant enhancements were observed
Kim et al. (112)	Effects of RAC on arm reaching performance in patients affected from stroke	7F, 9M (49.2 ± 17.6)	4	1.9 ± 2.2 years	Movement time, movement unit, elbow extension range of motion by 3D motion detection, triceps, biceps brachii muscle activation and co-contraction ratio from EMG	Repetitive reaching task performed with/without RAC from affected arm	RAC at patients preferred pace of movement	Significant enhancement in elbow range of motion, tricpes brachii activation with RAC Significant reduction in co-contraction ratio, movement time and movement unit with RAC
Shahine and Shafshak (113)	Effect of RAC on arm function in patients affected from stroke	Exp: 19F, 21M (61.4 ± 5.5) Ct: 17F, 19M (62.7 ± 3.1)	9	Exp: 2.6 ± 1.8 years Ct: 2.9 ± 0.7 years	FMA and transcutaneous magnetic stimulation eliciting motor evoked potential in paretic abductor pollicis brevis (motor evoked potential resting threshold, central motor conduction time)	Pre-test, BATRAC for 1-h session/day, 3seesions/week, for 8 weeks, post-test	RAC at patients preferred pace of movement (frequency 0.25–1/s)	Significant enhancement in FMA, motor evoked potential amplitude ratio after BATRAC Significant reduction in motor evoked potential resting threshold, central motor conduction time after bilateral arm training with RAC, and in Exp as compared to Ct
Dispa et al. (114)	Effect of RAC on arm reaching in patients affected from stroke	1F, 9M (66 ± 11.1)	8	2.3 ± 2.6 years	Grip lift parameters (preloading-loading phase, maximum grip force, hold ratio, cross correlation coefficient, time shift), digital dexterity, activity limitation manual ability, satisfaction in activities, and participation	Pre-test, post-test after 4 weeks of no-training, unilateral-bilateral (modified BBT) repetitive grip lift task oriented training with RAC for 1-h session, 3 days/week for 4 weeks, post-test at 4 weeks after training, retention measurement after 4 weeks	RAC at patients preferred pace of movement	Reduction in preloading phase of grip lift parameters for the paretic hand after 4 weeks of training and during retention measurement. Enhancement in loading phase of grip lift parameters for the paretic hand after 4 weeks of training and during retention measurement. No effect on grip lift parameters (maximum grip force, hold ratio, cross correlation coefficient, time shift), digital dexterity, activity limitation manual ability, satisfaction in activities, and participation after 4 weeks of training or during retention measurement.
Whitall et al. (115)	Effect of RAC in arm reaching patients affected from stroke	Exp: 16F, 26M (59.8 ± 9.9) Ct: 26F, 24M (57.7 ± 12.5) fMRI: Exp: 10F, 7M (61.2 ± 13.8) Ct: 10F, 11M (54.8 ± 13.1)	6	Exp: 4.5 ± 4.1 years Ct: 4.1 ± 5.2 years	FMA, wolf motor test (time, weight, function, SIS (emotion, hand, strength), isokinetic strength (elbow flexion-extension nonparetic side, elbow extension paretic side) and isometric strength (shoulder extension, wrist flexion-extension nonparetic side, shoulder extension, wrist extension, elbow flexion paretic side)	Pre-test, BATRAC for 1-h session, 3 times/week for 6 weeks, post-test	RAC at patients preferred pace of movement	Significant enhancement in FMA, wolf motor test (weight, function), SIS (hand, strength), isokinetic strength (elbow extension nonparetic side, elbow extension paretic side), isometric strength (shoulder extension, wrist extension nonparetic side, shoulder extension) assessment in Exp after training with RAC Significant reduction in wolf motor test (time) in Exp after training with RAC Significant enhancement in isokinetic strength (elbow flexion nonparetic side), isometric strength (wrist flexion, nonparetic side, wrist extension paretic side) in Exp as compared to Ct
		Exp: 3.9 2.7 Ct: 3.3 2.1						Significant enhancement in activation for ipsilesional precentral, anterior cingulate, postcentral gyri, supplementary motor area in Exp after training with RAC as compared to Ct. Significant enhancement in contralesional superior frontal gyrus in Exp after training with RAC, as compared to Ct
Chouhan and Kumar (116)	Effect of RAC on gait and arm reaching in patients affected from stroke	Exp: 3F, 12M (56.7 ± 5.9) Ct I: 3F, 12M (58.1 ± 4.1) Ct II: 3F, 12M (57.3 ± 5.5)	5	–	Dynamic gait index and FMA	Pre-test, gait, reaching task training with RAC (0% of preferred cadence initially, increased by +10% every week if comfortable for patient: for gait) (Exp) or visual feedback (Ct I) for 2 h training, 3 time/week session for 3 weeks, post-tests at 7, 14, 21, 28 days	RAC at 0% and +10% on following weeks of preferred movement pace, and gait (cadence)	Significant enhancements in FMA, dynamic gait index (14, 21, 28 days only) after 7, 14, 21, 28 days of training with RAC and in Exp as compared to Ct II
Secoli et al. (117)	Effect of RAC on tracking task in patients affected from stroke.	Exp: (affect left hemisphere) 8F, 6M (56.3 ± 12.3) Ct: (affect right hemisphere) 1F, 4M (61.8 ± 5) Healthy: 2F, 12M (27 ± 7.5)	4	4.6 ± 1 years	Arm movements with robot assisted force production to execute task in Z dimension Positioning error in Z dimension	Patients performed tracking task with robot assisted device in with/without visual distractor task and/or with/without RAC	Tonal beeps sampled at frequency of 800 Hz and lasting for 0.1 s Frequency manipulated proportionally to vector magnitude of position tracking error within dead-zone. Error direction determined by left and right channel of auditory input	Significant reduction in robot assisted force in Exp when auditory input was delivered, suggesting significant enhancement in arm functioning Significant reduction in robot assisted force for the paretic side as compared to healthy side with RAC No effect on position tracking accuracy
Thielman (118)	Effect of RAC on arm reaching in patients affected from stroke	Exp: 2F, 6M (62.9 ± 6.5) Ct: 4F, 4M (63 ± 9.2)	4	Exp: 2.2 ± 0.7 years Ct: 1.8 ± 1.4 years	Reaching performance scale for near and far targets, FMA, wolf motor function test, shoulder flexion range of motion, motor activity log, grip strength and elbow active range of motion	Pre-test (< 5days before training), training for arm reaching with pressure sensor generated auditory feedback (Exp), stabilizer (restrained Ct) on arm reaching for 40-45 minutes' session, 2-3days/week (12 total sessions), post-test (< 2days after training)	R-af (proportional to reduced pressure by shoulder on the sensor) Frequency faded off with progression from every 1/3^rd^ trial.	Significant enhancement in reaching performance scale for near-far targets, FMA in Exp after training with R-af Significant reduction in wolf motor function test in Exp after training with R-af Enhancement in shoulder flexion, motor activity log and elbow active range of motion in Exp after training with R-af
Johannsen et al. (119)	Effect of RAC on arm reaching and gait in patients affected from stroke	Exp I: 3F, 8M (59.5 ± 13.4) Exp II: 3F, 7M (68.1 ± 10.1)	6	5.2 ± 4.2 years	FMA (upper/lower extremity), 10-m walking test, treadmill (step length) and repetitive foot/hand aiming task	Pre-test, BATRAC (arm: Exp I/leg: Exp II) for 45 min session, 2 times/week for 5 weeks, post-test, follow up post-test after 18 weeks	RAC at patients preferred pace of movement (increased at patient's preference) Bilateral training for lower extremities: increased pacing during training from 36.7 ± 6.5 to 45.9 ± 9.5 beats per minute BATRAC: increased pacing during training from 39.8 ± 5.6 to 46.3 ± 5.9 beats per minute	Significant enhancement in treadmill step length on both paretic and non-paretic side after bilateral leg training in Exp II as compared to Exp I (no effects), during immediate follow-up test. No effects in follow up post-test. Enhancement in FMA test for lower extremity in Exp II> Exp I at post-test. No enhancements in follow up post-test Enhancement in fugl meyer motor test for upper extremity in Exp I> Exp II at post-test. No enhancements in follow up post-test Enhancement in treadmill step length on both paretic and non-paretic side after bilateral arm training in Exp I as compared to Exp II for 18 week follow up post-test Enhancement in repetitive foot and arm aiming task on both paretic and non-paretic side after bilateral leg training in Exp II during immediate post-tests. No effects on follow up post-tests
Stoykov et al. (120)	Effects of RAC on arm reaching in patients affected from stroke	Exp I: 3F, 9M (63.8 ± 12.6) Exp II: 5F, 7M (64.7 ± 11.1)	6	Exp I: 9.5 ± 5.4 years Exp II: 10.2 ± 10.1 years	Motor assessment scale (upper arm function, hand movements, upper limb items, advanced hand activities), motor status scale (total, shoulder-elbow, wrist-hand scale), shoulder flexion strength and wrist flexion-extension strength	Pre-test, arm reach training with bilateral (Exp I), unilateral (Exp II) arm training with RAC (for 4 tasks), for 60 minutes' session, 3 times/week for 8 weeks, post-test, RAC (rhythmic flexion-extension at the wrist joint)	RAC at patients preferred pace of movement (0.25-1.5 Hz) incremented gradually during training	Significant enhancement in motor assessment scale (upper arm function, upper limb items) for Exp I Significant enhancement in motor status scale (total, shoulder-elbow, wrist-hand scale), shoulder flexion strength, wrist flexion-extension strength for Exp I and Exp II Enhancement in motor assessment scale (advanced hand activities) in Exp I No differences in motor assessment scale for unilateral arm training for Exp II
Richards et al. (121)	Effects of RAC on arm reaching in patients affected from stroke	5F, 9M (64.4 ± 12.8)	4	5.4 ± 4 years	FMA, wolf motor function test and motor activity log (use, ability)	Pre-test, BATRAC for 1-hour session, 3 times/week, for 6 weeks, post-test	RAC at patients preferred pace of movement	Enhancement in FMA, motor activity log (ability and use) in Exp after training with RAC No effect on wolf motor function test in Exp after training with RAC
Jeong and Kim (122)	Effects of RAC on range of motion, flexibility in patients affected from stroke	Exp: 5F, 11M (58 ± 7.1) Ct: 5F, 12 M (62.2 ± 8.1)	4	Exp: 5.4 ± 4.5 years Ct: 7.2 ± 5.3 years	Shoulder flexion, ankle flexion-extension range of motion and back-scratch test for flexibility upwards/downward the affected arm, profile of mood states, relationship change scale and stroke specific quality of life scale	Pre-test, training for motor activities with RAC for 2 hours/ week for 8 weeks (functional ambulatory training), and self-training at home, post-test	RAC (music) at patients preferred pace of movement	Significant enhancement of range of motion for shoulder flexion, ankle flexion-extension, shoulder flexibility in Exp as compared to Ct, on the affected side Significant enhancement of mood states, interpersonal relationships in Exp Enhancement in quality of life in Exp
Waller and Whitall (123)	Effects of RAC on arm reaching in patients affected from stroke	Right hemisphere lesion: 2F, 9M (64.3 ± 10) Left hemisphere lesion: 3F, 8M (58.6 ± 17)	5	6.5 ± 4.1 years	FMA, University of Maryland questionnaire for stroke, wolf motor arm test (weight, time), active range of motion elbow flexion, shoulder extension and strength (shoulder extension-abduction-adduction, wrist flexion-extension)	Pre-test, BATRAC for 1-h session, 3 times/week for 6 weeks, post-test	RAC at patients preferred pace of movement	Significant enhancement in FMA, University of Maryland questionnaire, active range of motion elbow flexion, shoulder extension (left hemisphere only), strength (shoulder extension (left hemisphere only)-abduction (left only)-adduction, wrist flexion-extension), wolf motor arm test (weight) for stroke for right and left hemisphere lesion patients after training with RAC Significant reduction in wolf motor arm test (time) for patients with left hemisphere lesions as compared right hemisphere lesions after training with RAC Significant enhancement in University of Maryland questionnaire for stroke, wolf motor assessment test (weight, time), active range of motion elbow flexion, shoulder extension, strength (shoulder extension-abduction-adduction, wrist flexion-extension) for patients with left hemisphere lesions as compared to right hemisphere lesions after training with RAC
Luft et al. (95)	Effect of RAC on arm function in patients affected from stroke	Exp: 2F, 7M (63.3 ± 15.3) Ct: 7F, 5M (59.6 ± 10.5)	6	6.2 (3.1–7) years	FMA, shoulder, elbow strength, Wolf motor arm test (weight, time), University of Maryland arm questionnaire for stroke and functional magnetic resonance imaging	Pre-test, BATRAC for 1-h session/day, 3 times/week for 6 weeks, post-test	RAC at patients preferred pace of movement (0.67–0.97 Hz)	After exclusion of 3 patients from Exp: Significant enhancement in FMA in Exp as compared to Ct. Enhancement in shoulder, elbow strength, Wolf motor arm test (weight), University of Maryland arm questionnaire for stroke in Exp as compared to Ct. Reduction in wolf motor arm test (time) in Exp as compared to Ct. Significant enhancements in cerebellum, precentral and postcentral gyri activation after BATRAC
Thaut et al. (124)	Effects of RAC on arm reaching task in patients affected from stroke	8F, 13M (52.7 ± 13.7)	4	–	Wrist trajectory, elbow, shoulder kinematic and optimization model of peak acceleration of wrist joint coordinates	Reaching tasks initiated with/without rhythmic auditory feedback/external time cueing (counterbalanced)	RAC at patients preferred pace of movement 1,000 Hz square wave tone 50 ms pattern	Significant enhancement in elbow range of motion with RAC Significantly reduced trajectory variability of wrist joint, deviation of acceleration curves from optimal coordinates of wrist joint with RAC No effect on arm timing, shoulder joint displacement
Maulucci and Eckhouse (125)	Effects of R-af on reaching task in patients affected from chronic stroke	Healthy: 15F, 9M Exp: 3F, 5M Ct:4F, 4M	4	–	Normal trajectory region for end effectors and reach parameters	Normal participants performed and established generalized repeatability for the experimental groups. Reach trials performed for 42 trials 3 times/week for 6 weeks. Residual performance evaluated post 2 weeks without auditory feedback	Regulated R-af of magnitude and existence of error from normal ellipsoid reach area.	Significant enhancement in trajectory performance for Exp group as compared to Ct group Significant enhancement in both Exp and Ct group for reach trajectory
Whitall et al. (126)	Effects of RAC on arm motor function in patients affected from stroke	6F, 8M (63.7 ± 12.6)	6	5.5 ± 7.9 years	Active, passive range of motion of upper extremity, isometric shoulder, elbow, wrist force (flexion/extension) assessment, FMA, wolf motor function test and modified University of Maryland arm questionnaire for stroke	Pre-test, BATRAC for four 5-min sessions, 3 times a week for 6 weeks, post-test, 8-week retention post-test	RAC at patients preferred pace of movement	Significant enhancement in FMA, Wolf motor function test and modified University of Maryland arm questionnaire for stroke with RAC Significant enhancement in elbow, wrist flexion for paretic and non-paretic arm with RAC Significant enhancement in active range of motion for shoulder extension, wrist flexion and thumb opposition and passive range of motion for wrist flexion on the paretic side with RAC Significant enhancements sustained during the 8-week retention post-test across all range of motion, strength variables and qualitative assessment tools with RAC

*ARAT, Action reach arm test; 9HPT, 9-hole peg test; FMA, Fugl Meyer assessment for upper extremity; BBT, Box and block test; EMG, Electromyography; RAC, Rhythmic auditory cueing; BATRAC, Bilateral arm training with rhythmic auditory cueing; R-af, Real-time auditory feedback; SIS, Stroke impact scale; Exp, Experimental group; Ct, Control group; F, Females; M, Males*.

The inclusion criteria for the studies was (i) The experimental studies were either randomized controlled trials, cluster randomized controlled trials or controlled clinical trials; (ii) The included studies reported reliable and valid measures to analyse arm function, and/or kinematic parameters; (iii) The included studies analyzed subjective analysis of stroke outcome; (iv) The included studies scored ≥4 score on the PEDro methodological quality scale; (v) The experiments conducted on human participants; (vi) The included studies were published in a peer-reviewed academic journal, conference proceeding; (vii) The included studies were published in English, Hindi, Punjabi, and German languages.

### Quality and risk of bias assessment

The quality of the included experimental studies was assessed using the PEDro methodological quality scale (127). This scale consists of 11 items which address both external, internal validity. Moreover, its interpretation can effectively detect potential bias with fair to good reliability, and validity (127). A blinded scoring for the methodological quality was carried out by the primary reviewer (S.G). If any ambiguous issues were there concerning rating of the studies. These issues were discussed with a second reviewer (Dr. Ishan Ghai). Included studies were interpreted according to a scoring of 9–10, 6–8, and 4–5 considered as “excellent,” “good,” and “fair” quality, respectively (128).

### Data analysis

For a better interpretation of the intervention effects, a meta-analysis was included (129). The absence of presence of heterogeneity asserted the use of either fixed or random effect meta-analysis (130), respectively. A narrative synthesis of the findings structured around the intervention, population characteristics, duration of stroke, auditory signal characteristics, methodological quality, and type of outcome are provided (Table 2). A meta-analysis was conducted between pooled homogenous studies using CMA (Comprehensive meta-analysis V 2.0, USA). Heterogeneity between the pooled studies was assessed and interpreted using I^2^ statistics. The data in this present review was systematically distributed and pooled for each variable. Thereafter, forest plots with effect size and 95% confidence intervals were plotted. The effect sizes were weighted and reported as Hedge's g (131). Thresholds for interpretation of effect sizes are as follows; a standard mean effect size of 0 meant no intervention effect, negative effect size meant a negative intervention effect, and a positive effect size meant a positive intervention effect. Further, a mean effect size of 0.2 was interpreted as a *small* effect, 0.5 interpreted as a *medium* effect, and 0.8 interpreted as a *large* effect (132). Interpretation of heterogeneity made from I^2^ statistics was as following: 0–0, 25, 75% was interpreted as negligible, moderate, and substantial heterogeneity, respectively. The alpha level was set at 95%.

## Results

### Characteristics of included studies

**Figure 1:**
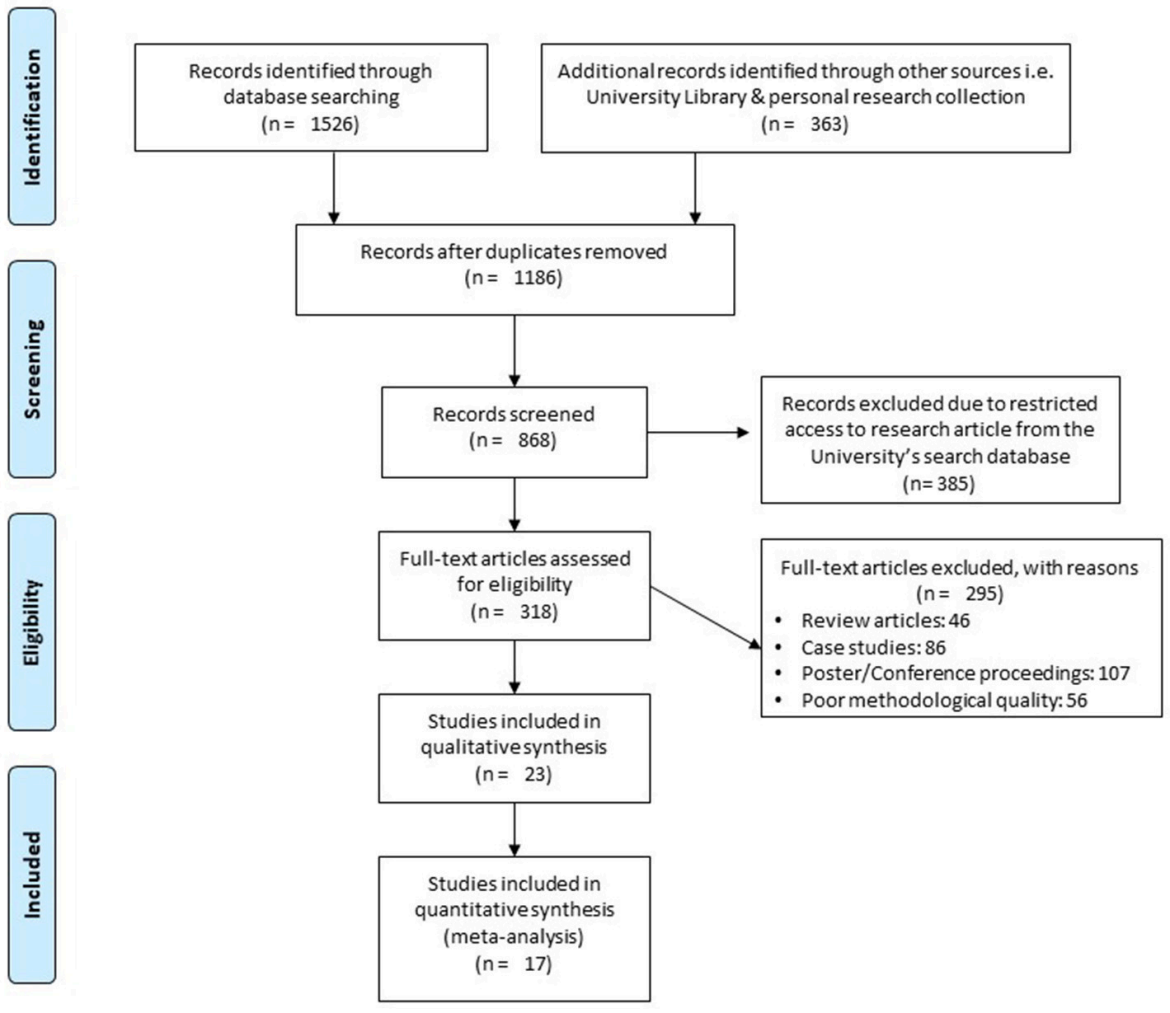
PRISMA flow chart for the inclusion of studies.

A detailed search criterion has been demonstrated in Figure 1. Out of 1,889 studies, only 23 studies qualified our inclusion criteria. A total of 385 studies could not be included in the manuscript due to limitations in access by University's search database. The author (S.G) made attempts to contact the respective corresponding authors for retrieving the manuscripts. Although these studies could not be included in the review, the abstracts for all the studies were individually screened by the reviewers. The reviewers did not find any counterbalancing data. Data from each included study has been summarized in (Table 2). In the included studies, 10 were randomized controlled trials, and 13 were controlled clinical trials. Interventions in all the included studies were performed by either physiotherapists or medical practitioners. However, two studies in addition to training in clinics/laboratories included a phase of self-training administered by the patients themselves, at home (108, 122). Here, in both the studies guidance was provided by the researchers to the patients via telephone.

### Participants

In total, the 23 included studies evaluated 585 participants of mixed gender population. The included studies had the gender distribution as follows: 226 females, and 359 males. Descriptive statistics concerning age (mean ± standard deviation) of the participants were tabulated across the studies. Disease duration of stroke patients has also been mentioned for better interpretation of the reader. However, five studies did not mention these details (107, 109, 111, 124, 125).

### Risk of bias

**Figure 2:**
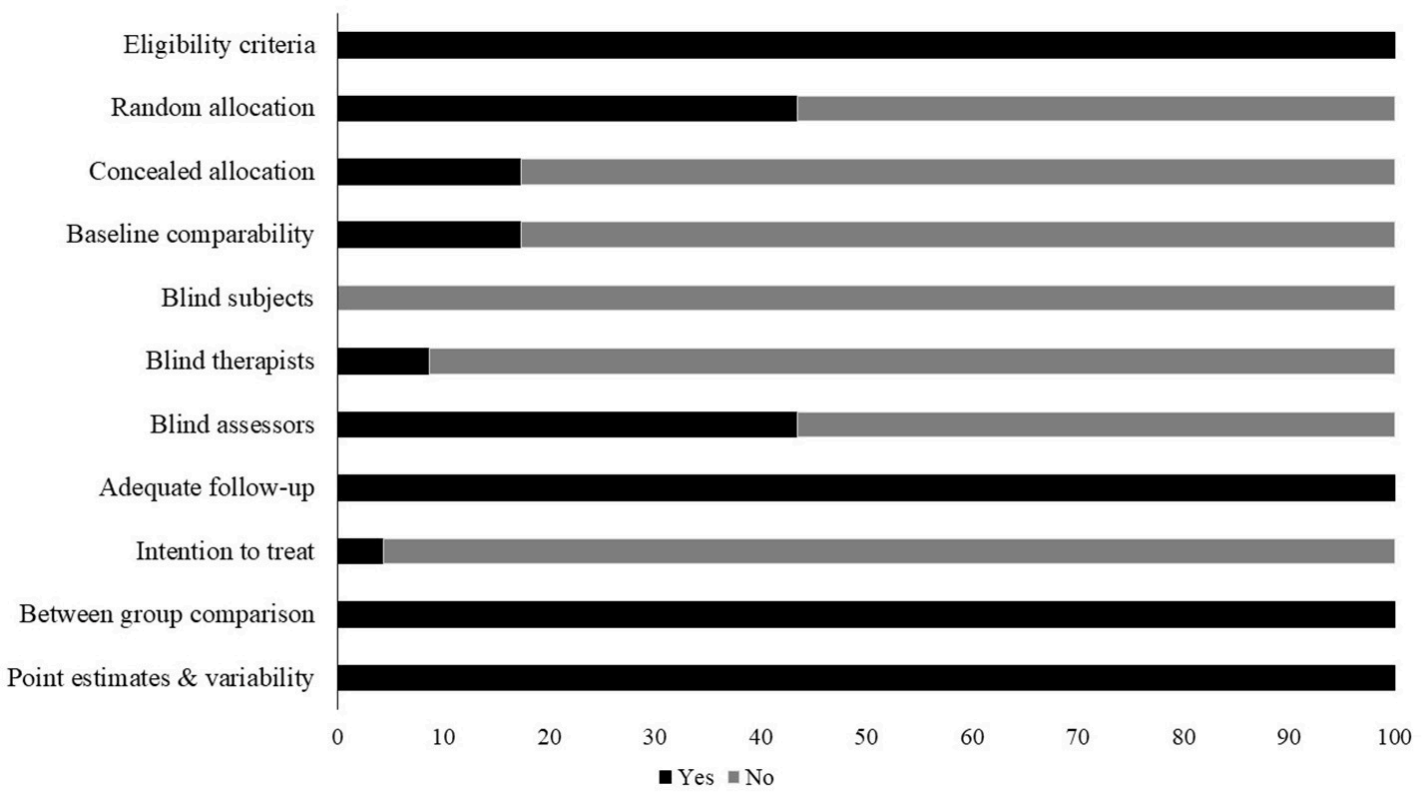
Risk of bias across studies.

**Figure 3:**
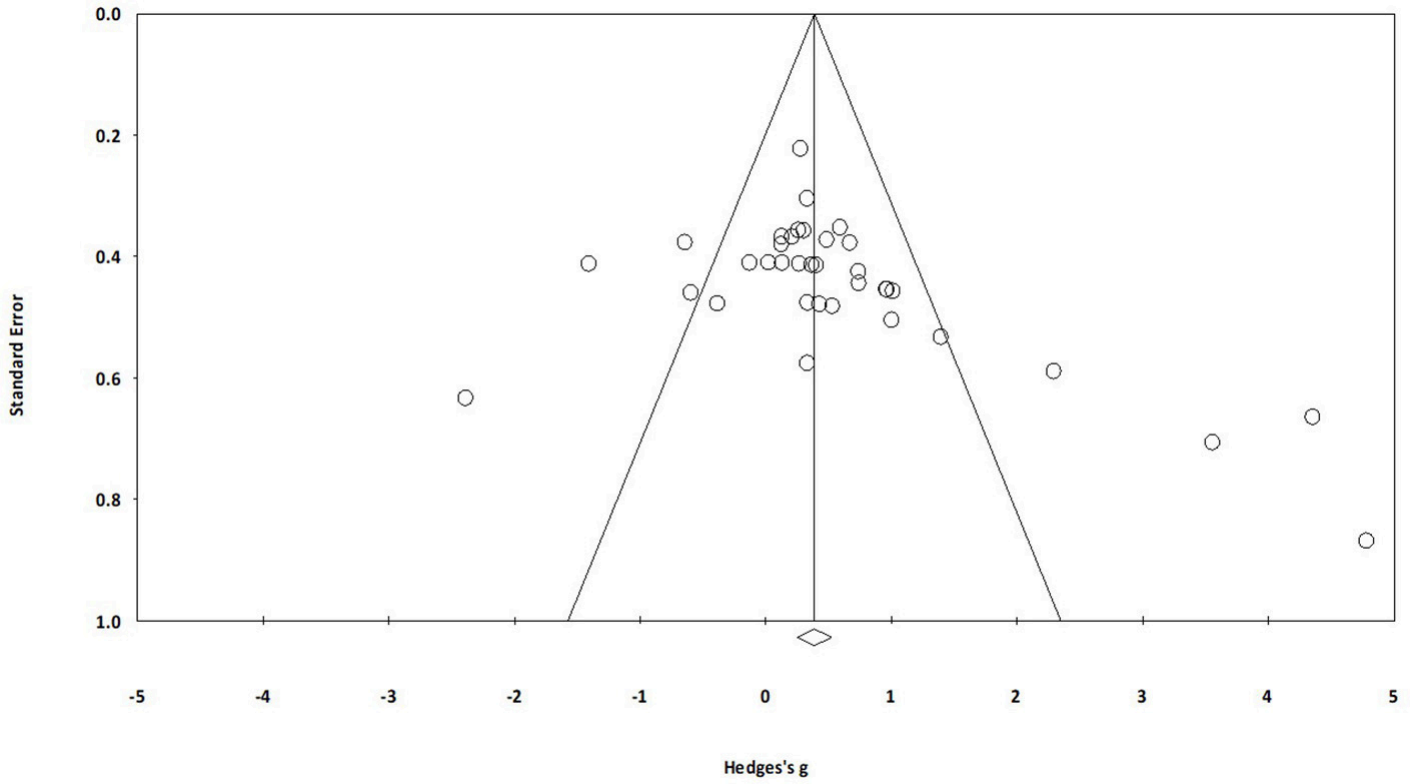
Funnel plot for Hedge's g and standardized effect for each value in the meta-analysis. Each of the effect is represented in the plot as a circle. Funnel boundaries represent area where 95% of the effects are expected to lie if there were no publication biases. The vertical line represents the mean standardized effect of zero. Absence of publication bias is represented by symmetrical distribution of effect's around the mid-line.

Studies scoring ≥4 on PEDro methodological scale were included in the review. Individual scores have been reported (Table 2, Supplementary Table 1). The average PEDro score for the 23 included studies was computed to be 5.3 ± 1.6 out of 10, indicating “fair” quality of the overall studies. Here, two studies scored nine (excellent quality), one study scored eight (excellent quality), three studies scored seven (good quality), six studies scored six (good quality), two studies scored five (fair quality), and 11 studies scored four (fair quality) (Table 2, Supplementary Table 1). Figure 2 illustrates risk of bias across the studies. Further, publication bias was analyzed by plotting the evaluated weighted effect size i.e., Hedge's g values against standard error (Figure 3). Here, any asymmetry concerning mean in the funnel plot might suggest the presence of publication related bias.

### Meta-analysis

#### Outcomes

The results clearly suggest a positive influence of training with rhythmic auditory cueing and real-time auditory feedback on arm recovery post-stroke. Out of 23 included studies, significant enhancement was reported in 19 studies, three studies reported enhancements, and only one study reported significant reduction in arm function post training with auditory stimuli (Table 2).

### Meta-analysis report

Application of a strict inclusion criterion was also meant to limit the amount of heterogeneity between the pooled studies (133). Nevertheless, despite these attempts some amount of unexplained heterogeneity was still observed. Thereafter, attempts were made to pool and analyze the studies further in sub-groups. The meta-analysis evaluated arm-functioning parameters, such as Fugl-Meyer assessment scores, Wolf motor time test, Action reach arm test, Stroke impact scale, 9-hole peg test, and elbow range of motion. The reliability and validity of these tests has been proven in the literature (134). Further, sub-group analyses were conducted to analyze specific training dosages, and to compare the effects of rhythmic auditory cueing and real-time auditory feedback. The main reasons for excluding the studies from statistical analysis was either major differences in between assessment methods, for instance considerably different auditory stimuli, disease duration, and/or lack of descriptive statistics within the manuscript. In this case, attempts were made by the primary reviewer (S.G) to contact respective corresponding authors.

### Fugl meyer assessment score

**Figure 4:**
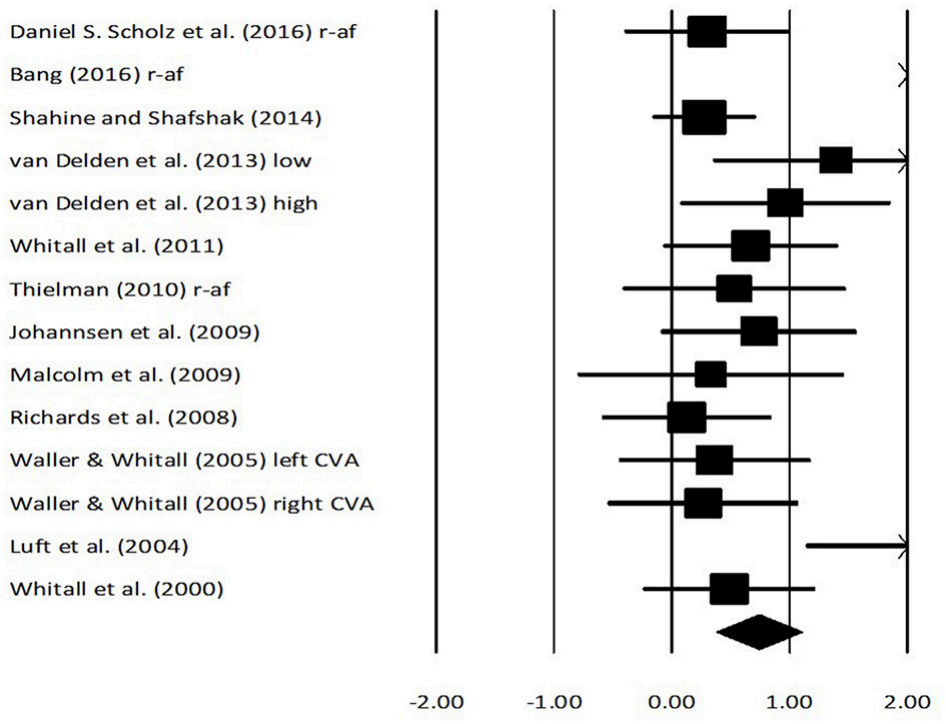
Forest plot illustrating individual studies evaluating the effects of rhythmic auditory cueing, and real-time auditory feedback on Fugl Meyer assessment scores on arm function amongst post stroke patients. Weighted effect sizes; Hedge's g (boxes) and 95% C.I (whiskers) are presented, demonstrating repositioning errors for individual studies. The (Diamond) represents pooled effect sizes and 95% CI. A negative effect size indicated reduction in Fugl Meyer scores depicting poor arm functioning; a positive effect size indicated enhancement in Fugl Meyer scores depicting better arm functioning. (r-af, Real-time auditory feedback; low, Low performance group; high, High performance group; left CVA, Left sided cerebrovascular accident; right CVA, Right sided cerebrovascular accident).

Fugl Meyer assessment scores for arm performance were assessed in 11 studies. Here, two studies evaluated the score on stroke patients while using real-time auditory feedback, whereas nine studies utilized rhythmic auditory cueing. The analysis of studies revealed (Figure 4) a *large* effect size in the positive domain (g: 0.79, 95% C.I: 0.38–1.09) and moderate heterogeneity was observed in between the studies (*I*^2^: 29.3%, *p* > 0.05). Further, on separating the studies for comparing the effects of rhythmic auditory cueing and real-time auditory feedback, nine studies were analyzed for their effects on rhythmic auditory cueing and three studies for real-time auditory feedback.

An analysis for effects of rhythmic auditory cueing on Fugl Meyer assessment revealed (Supplementary Figure 1), positive *medium* effect size with negligible heterogeneity (g: 0.6, 95% C.I: 0.30–0.91, *I*^2^: 10.7%, *p* > 0.05). An analysis for effects of real-time auditory feedback on Fugl Meyer assessment revealed (Supplementary Figure 2), a larger positive *large* effect size with moderate heterogeneity (g: 1.3, 95% C.I: −0.25 to 2.8, *I*^2^: 40.3%, *p* > 0.05).

A further sub-group analysis based on the amount of training dosage (30 min to 1 h, ≥3 sessions per week) for rhythmic auditory cueing revealed (Supplementary Figure 3), positive *medium* effect size with moderate heterogeneity (g: 0.54, 95% C.I: 0.3–0.78, *I*^2^: 43.8%, *p* = 0.06). Only one study (126), performed a training with rhythmic auditory cueing for < 30 min, and hence was not included in further analysis. For the real-time auditory feedback Supplementary Figure 2 also illustrates the effects of training dosage for 30–45 min per session, and for >10 sessions of training.

### Wolf motor time assessment

**Figure 5:**
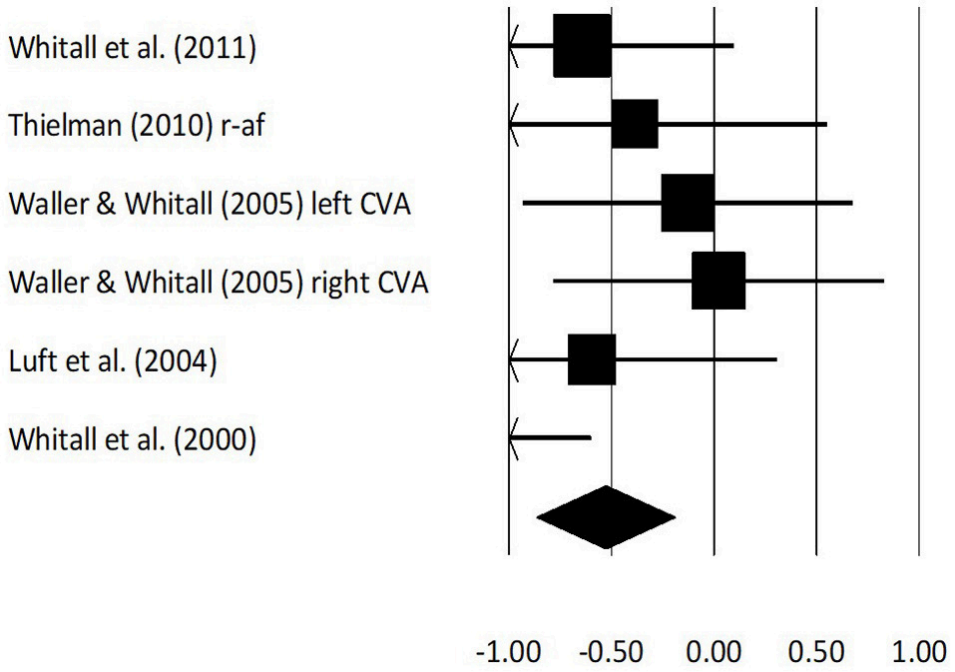
Forest plot illustrating individual studies evaluating the effects of rhythmic auditory cueing, and real-time auditory feedback on Wolf motor time assessment scores for arm function amongst post stroke patients. Weighted effect sizes; Hedge's g (boxes) and 95% C.I (whiskers) are presented, demonstrating repositioning errors for individual studies. The (Diamond) represents pooled effect sizes and 95% CI. A negative effect size indicated reduction in Wolf motor scores depicting a better arm functioning; a positive effect size indicated enhancement in Wolf motor scores depicting poor arm functioning. (r-af, Real-time auditory feedback; low, Low performance group; high, High performance group; left CVA, Left sided cerebrovascular accident; right CVA, Right sided cerebrovascular accident).

An analysis for effects of rhythmic and real-time auditory stimuli on Wolf motor time assessment revealed (Figure 5) a negative *medium* effect size with moderate heterogeneity (g: −0.52, 95% C.I: −0.86 to −0.19, *I*^2^: 33.2%, *p* = 0.18). Further, an analysis for only rhythmic auditory cueing revealed (Supplementary Figure 4) a similar negative *medium* effect size with negligible heterogeneity (g: −0.55, 95% C.I: −1.04 to −0.05, *I*^2^: 0%, *p* > 0.05).

A further sub-group analysis based on the amount of training dosage (30 min to 1 h, ≥3 sessions per week) for rhythmic auditory cueing revealed (Supplementary Figure 5), negative *medium* effect size with negligible heterogeneity (g: −0.34, 95% C.I: −0.71 to 0.02, *I*^2^: 0%, *p* > 0.05).

### Elbow range of motion

**Figure 6:**
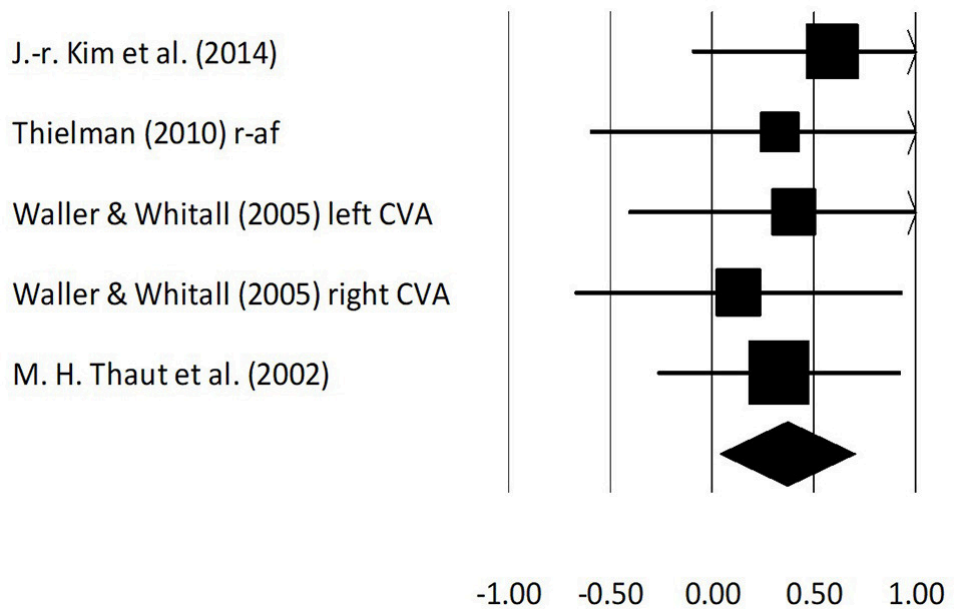
Forest plot illustrating individual studies evaluating the effects of rhythmic auditory cueing, and real-time auditory feedback on elbow range of motion among post stroke patients. Weighted effect sizes; Hedge's g (boxes) and 95% C.I (whiskers) are presented, demonstrating repositioning errors for individual studies. The (Diamond) represents pooled effect sizes and 95% CI. A negative effect size indicated reduction in elbow range of motion depicting poor arm functioning; a positive effect size indicated enhancement in elbow range of motion depicting better arm functioning. (r-af, Real-time auditory feedback; low, Low performance group; high, High performance group; left CVA, Left sided cerebrovascular accident; right CVA, Right sided cerebrovascular accident).

Analysis for effects of rhythmic and real-time auditory stimuli on elbow range of motion revealed assessment revealed (Figure 6) a positive *medium* effect size with negligible heterogeneity (g: 0.36, 95% C.I: 0.03–0.7, *I*^2^: 0%, *p* > 0.05). Further, a sub-group analysis for only rhythmic auditory cueing revealed a similar positive *medium* effect size with negligible heterogeneity (g: 0.37, 95% C.I: 0.01–0.72, *I*^2^: 0%, *p* > 0.05). Further sub-group analysis was not performed because two studies did not include a training regime (112, 124), and one study analyzed the effects of real-time auditory feedback (118).

### Action reach arm test

Analysis for effects of rhythmic and real-time auditory inputs on Action reach arm test revealed (Supplementary Figure 6) a positive *large* effect size with substantial heterogeneity (g: 0.95, 95% C.I: 0.49–1.42, *I*^2^: 87%, *p* = 0.01). Further, a sub-group analysis for only real-time auditory feedback training (30–45 min per session, and for >10 sessions of training) revealed a similar positive *large* effect size with substantial heterogeneity (g: 0.91, 95% C.I: 0.26–1.55, *I*^2^: 95.6%, *p* = 0.001). Here, heterogeneity could be affirmed to considerable differences in the characteristics of real-time auditory feedback provided to the patients (see Table 2 for details in auditory signal characteristics).

### Nine-hole peg test

Analysis for effects of rhythmic and real-time auditory stimuli on Nine-hole peg test revealed (Supplementary Figure 7) a positive *small* effect size with substantial heterogeneity (g: 0.12, 95% C.I: −0.32 to 0.58, *I*^2^: 85.2%, *p* = 0.01).

Further, a sub-group analysis for only rhythmic auditory cueing training (>30 min training session, 3 sessions per week) revealed a similar positive *small* effect size with substantial heterogeneity (g: 0.12, 95% C.I: −0.32 to 0.58, *I*^2^: 90.15%, *p* = 0.001). Here, heterogeneity could be affirmed to considerable differences in the characteristics of rhythmic auditory cueing provided to the patients (Table 2).

### Stroke impact scale

Analysis for effects of rhythmic and real-time auditory stimuli on Stroke impact scale revealed (Supplementary Figure 8) a positive *large* effect size with substantial heterogeneity (g: 0.95, 95% C.I: 0.49–1.42, *I*^2^: 87%, *p* = 0.01). Further, a sub-group analysis for only rhythmic auditory cueing (>30 min of training, 3 sessions per week) revealed a similar positive *large* effect size with substantial heterogeneity (g: 0.91, 95% C.I: 0.26–1.55, *I*^2^: 95.6%, *p* = 0.001). Here, substantial amount of heterogeneity could be due to considerable differences in the characteristics of real-time auditory feedback provided to the patients (Table 2).

## Discussion

The objective of this systematic review and meta-analysis was to analyze the current state of knowledge for the effects of rhythmic auditory cueing and real time kinematic auditory feedback for recovering arm function post-stroke. The current meta-analysis reports beneficial *small*-to-*large* standardized effects for both rhythmic auditory cueing and real-time kinematic auditory feedback in this aspect. Normally, patients with stroke exhibit poor spatiotemporal parameters during gross and fine motor skills performance for the upper extremities (135). Research suggests that assessment of arm function from Fugl Meyer test (136), Wolf motor assessment (137), Action reach arm test (138), 9-hole peg test (139), reliably reveal the severity of gross and fine motor function impairment post-stroke (136). In the current meta-analyses, we report beneficial effects of rhythmic auditory cueing on Fugl Meyer test (g: 0.6), Action reach arm test (g: 0.95), Wolf motor time test (g: −0.55), elbow range of motion (0.37), Nine-hole peg test (0.12), and Stroke impact scale (g: 0.91). Similarly, beneficial effects of real-time auditory feedback have also been reported for Fugl Meyer test (1.3), and action reach arm test (0.91). Therefore, indicating beneficial effects of external auditory stimuli for enhancing arm recovery, quality of life post-stroke.

Several reasons ranging from physiological, psychological and cognitive domains can be asserted for the beneficial effects of auditory stimuli on motor performance (64, 67, 83, 140, 141). Firstly, from a neurophysiological aspect, the auditory stimuli could have mediated multifaceted benefits. First and foremost, the stimuli could have facilitated or bypassed the deficit internal cueing system, often impaired in stroke patients exhibiting movement disorders (12). Here, a direct stimuli could have bypassed the deficit putamen directly to thalamus, and then from pre-motor area directly to primary motor cortex (76, 142). Secondly, the external stimuli could have modulated the oscillatory pattern of neuromagnetic β waves (a functional measure of auditory motor coupling) in auditory cortex, cerebellum, inferior frontal gyrus, somatosensory area and sensorimotor cortex (88, 143). Thirdly, enhanced neurological activation in inferior colliculi, cerebellum, brainstem, and sensorimotor cortex post training with rhythmic auditory cueing could have enhanced motor performance. In addition, enhanced neural re-organization especially in cortico-cerebellar circuits, and phase-periodic corrections (144) could have also been important reasons for enhancements in upper limb motor performance. Similarly, external auditory stimuli have also been suggested to facilitate neural plasticity (89, 96). In the present meta-analysis, we report beneficial effects of a training duration of 30 min−1 h with rhythmic and real-time auditory stimuli to result in enhanced performance measures for upper arm. According to the results of, this seems rational. The authors in their research reported enhanced electroencephalographic co-activity in the right hemispheric regions after just 20 min of audio-motor training, thereby implying a timeline for instigating plasticity (96). The authors also suggested the necessity of such time frame for establishing links between the perceptual modalities. Additionally, bilateral training could have also played an integral role in facilitating recovery observed in most of the studies (145). This training strategy has also been reported to facilitate neuroplasticity, cortical reorganization (110). Research suggest that bilateral training can facilitate plasticity by increasing bi-hemispheric activation, disinhibiting motor cortex, and upwardly regulating the descending propriospinal neurons.

In addition to these changes, the external auditory stimuli could also mediate debilitating cognitive dysfunctions commonly observed in patients with stroke (49). Published literature has often reported a direct relationship between the cognitive decline and movement failure (46, 146, 147). Masters and Maxwell (48) suggested that a cognitive decline might predispose patients to internally monitoring their movement patterns. This could then cause interferences with the autonomic functioning of the neural pathways, and might result in information overload (46), which further could lead to movement failure. Here, two explanations have been suggested in literature to counteract this cognitive overload. Firstly, the external auditory stimuli have been suggested to act as an external distractor (148). This could have allowed the patient to direct their focus away from their movements, thereby enhancing automatic control. Choi et al. (149) for instance, analyzed static and dynamic balance in chronic stroke patients during a cognitive-motor dual task. Here, the authors reported balance improvements when auditory cues were used during the dual task. The authors suggested that auditory cues might induce appropriate attention allocation i.e., engage higher attentional resources during auditory perception, which then could have facilitated motor performance. Secondly, enhanced cross modal processing between auditory and proprioceptive signals due to their high spatiotemporal proximity could have circumvented information overload in the native sensory modality by directing task-irrelevant information toward the underused sensory modality (98, 150). Here, inferences can be drawn from the Multiple resource theory (151, 152). The theory states that separate pools of attentional resources exist for different sensory channels and processes. Therefore, utilizing congruent stimuli together through different sensory modalities might reduce attentional interference by distributing the load amongst both the utilized modalities. Research analyzing the influence of cross-modal cueing between sensory modalities for instance audio-tactile domain have reported significant enhancements in performance under dual-task conditions as compared to performances under single sensory modality (150, 153) [for a detailed meta-analysis see (154)].

Moreover, recent research also suggests that in addition to mediating cognitive overload in patients with stroke, the external auditory cueing via music might facilitate, reorganize deficit cortical structures (155–157). For instance, merging the external auditory stimuli with music can allow facilitation of neural network including prefrontal, and limbic cortex this in turn has been associated with cognitive and emotional recovery post-stroke (155). Future research is strongly recommended to address this gap in literature as it might allow in developing of a rehabilitation protocol that focuses not only on motor recovery but also neural re-generation and/or organization (158).

In addition to the cognitive and motor deficits, the external auditory stimuli can also mediate lower sensory perceptual thresholds exhibited in patients in stroke (35). Here, external auditory stimuli might enhance the saliency of the perceptual modalities, which could then support the development of feedback, and feedforward models necessary for motor planning and execution (82, 159–161). Also, cross-sensory impacts between the perceptual modalities due to high spatiotemporal proximity between the sensory modalities might result in the auditory stimuli to support the deficit proprioceptive modality (98). Recent research evaluating the rhythmic auditory cueing suggests that mediating the auditory signal characteristics in terms of ecologically valid action relevant sounds might further enrich the precepted spatio-temporal information and allow extended enhancements in motor execution (142, 162) i.e., as compared to isosynchronous cueing. Patients with stroke due to their sensory impairments usually have higher thresholds for perception of sensory stimuli (35, 163). Therefore, enhancing the saliency of sensory information delivered through ecologically valid action relevant auditory stimuli such as walking on gravel, snow might be beneficial (50, 142, 164). According to Young et al. (165) action relevant auditory stimuli not only specify the temporal but also the spatial information, thereby enriching the feed-forward mechanisms to execute a motor task efficiently (166). The authors also affirmed beneficial effects of action relevant auditory stimuli on gait performance due to putative function of “sensori-motor neurons” (166). Furthermore, it can be expected that modifications in auditory signal characteristics such as modulation of timbre at a higher intensity further merged with a broad ascending melody and rich harmony might motivate a stroke patient to exert more force (50, 142, 167). This however, was not evaluated in any of the studies included in this review and should be a possible topic of research for future studies.

Moreover, research suggests the extended benefits of real-time auditory feedback with respect to rhythmic auditory stimuli. suggested that mapping the movements with real-time auditory feedback could allow a patient to better perceive their self-generated movement amplitudes. Further allowing them to compare it with the sound of a desirable auditory movement model. This could then result in development of an auditory reference framework model, which could amplify internal simulations of movements, and allow a patient to better perceive spatio-temporal parameters as compared to discrete rhythmic component (168). A contextual comparison of neuroimaging data from rhythmic (85, 86), and real-time auditory stimuli (90), suggests a large number of neurological structures having overlapped activation between both the auditory stimuli. However, enhanced activation of the areas associated with action observation such as, superior temporal sulcus, premotor cortex (169, 170), have been reported with real-time auditory feedback in one study (90). Here, the main reasons for the enhanced activation in areas associated with motion perception can be attributed to the findings of Shams and Seitz (171) and Lahav et al. (172). Here, the authors suggested that a convergent audio-visual motion would enhance accuracy of perception and motor performance due to the enhanced multimodal congruent nature (90, 171). Further, Lahav et al. (172) hypothesized that an audio-visual mirror neuron system with the premotor areas might be involved in serving as an “action listening” and “hearing & doing mirror neuron system,” with the latter being largely dependent on a person's motor repertoire. Likewise, Vinken et al. (173) demonstrated that mapping real-time auditory feedback with real life activities lead to enhanced accuracy in judgement of actions, thereby demonstrating enhanced potential for improving motor perception, control, and learning. In the present meta-analysis enhanced scores for Fugl Meyer scores with real time kinematic auditory feedback (g: 1.3) were observed as compared to rhythmic auditory cueing (0.60).

The auditory stimuli could have also influenced the musculoskeletal structure of the upper extremities. For example, research suggests that intricate neuroanatomical interconnections between the auditory and motor cortex could allow the auditory stimuli could possibly mediate the firing and recruitment rate of motor units (28). This could then result in smoothening of motor movements, further resulting in enhanced joint kinematics, and movement scaling parameters (174). Likewise, regularized muscle co-activation rate has also been documented in electromyographic studies (175–177). This was also demonstrated in our meta-analysis concerning enhancement in elbow range of motion with rhythmic auditory cueing.

Moreover, the application of these interventions can be promoted in a cost-effective manner due to their high viability (50, 142). The strategies could prove to be efficient in developing countries where higher costs of rehabilitation promote stroke associated morbidity and mortality (178, 179). Here, the medical practitioners or tele-stroke (179), helplines can promote the use of mobile applications which can be utilized by patients at their home. Few smartphone applications have been reported in published literature, however, their feasibility in terms of costs is too high (180, 181). Future studies are recommended to address this gap and develop open source applications for the use of stroke patients. Here, the global position sensors, gyroscope and accelerometers present usually in a smartphone can be utilized to direct kinematic information, which could then assist in projecting either optimal rhythmic cueing pattern or converted/mapped in real-time to produce sonified auditory feedback. Further, applications can be developed to generate different types of ecologically valid sounds.

Finally, as the current review mentions a sole author (S.G), concerns regarding biasing, methodological flaws in the study's design and outcomes could be expected (182). Here, the reader is assured that this present systematic review and meta-analysis was carried out by two authors. Dr. Ishan Ghai (I.G) acted as an additional reviewer and statistician in the current study. His role is duly mentioned in the methodological, and acknowledgment sections. Dr. Ishan Ghai has himself consented to be excluded from this study as a co-author. Moreover, to ensure transparency in the methodological parts of the current review and analyses sufficient description has been provided for reciprocating the search strategy (Table 1), and the statistical analysis. Additionally, the corresponding author is willing to share the entire data with any reader upon request.

In conclusion, this present review for the first time analyzed the effects of rhythmic and real time auditory stimuli on arm recovery in post-stroke patients. The present findings are in agreement with systematic reviews and meta-analysis carried out to analyze auditory entrainment effect on aging (50), cerebral palsy (164), stroke (183), multiple sclerosis (184), and parkinsonism (63, 185). This review strongly suggests the incorporation of rhythmic and real-time auditory stimuli with a training dosage of 30 min to 1 h of training, for >3 sessions week for enhancing arm function recovery post-stroke.

## Author contributions

SG conceptualized the study, carried out the systematic review, statistical analysis, and wrote the paper.

### Conflict of interest statement

The author declares that the research was conducted in the absence of any commercial or financial relationships that could be construed as a potential conflict of interest.
