# Supplementary material for: Effects of Real-Time (Sonification) and Rhythmic Auditory Stimuli on Recovering Arm Function Post Stroke: A Systematic Review and Meta-Analysis
Source: Front Neurol. 2018 Jul 13;9:488. doi: 10.3389/fneur.2018.00488 (PMC6053522; doi:10.3389/fneur.2018.00488)
Supplement: Supplementary file 1 [file Data_Sheet_1.DOCX]

## Effects of real-time (sonification) and rhythmic auditory stimulations on recovering arm function post stroke: A systematic review & meta-analysis

Shashank Ghai

Supplementary File

Table 1 Individual Pedro scores for studies (1: point awarded, 0: no point awarded)

| Study | PEDRO | Point estimates & variability | Between group comparison | Intention to treat | Adequate follow-up | Blind assessors | Blind therapists | Blind subjects | Baseline comparability | Concealed allocation | Random allocation | Eligibility criteria |
| --- | --- | --- | --- | --- | --- | --- | --- | --- | --- | --- | --- | --- |
| Bang [1] | 9 | 1 | 1 | 0 | 1 | 1 | 1 | 0 | 1 | 1 | 1 | 1 |
| Scholz *et al.* [2] | 4 | 1 | 1 | 0 | 1 | 0 | 0 | 0 | 0 | 0 | 0 | 1 |
| Malcolm *et al.* [3] | 4 | 1 | 1 | 0 | 1 | 0 | 0 | 0 | 0 | 0 | 0 | 1 |
| Speth [4] | 4 | 1 | 1 | 0 | 1 | 0 | 0 | 0 | 0 | 0 | 0 | 1 |
| Scholz *et al.* [5] | 7 | 1 | 1 | 0 | 1 | 0 | 0 | 0 | 1 | 1 | 1 | 1 |
| van Delden *et al.* [6] | 4 | 1 | 1 | 0 | 1 | 0 | 0 | 0 | 0 | 0 | 0 | 1 |
| Schmitz *et al.* [7] | 4 | 1 | 1 | 0 | 1 | 0 | 0 | 0 | 0 | 0 | 0 | 1 |
| Kim *et al.* [8] | 9 | 1 | 1 | 0 | 1 | 1 | 1 | 0 | 1 | 1 | 1 | 1 |
| Shahine and Shafshak [9] | 8 | 1 | 1 | 0 | 1 | 1 | 0 | 0 | 1 | 1 | 1 | 1 |
| Dispa *et al.* [10] | 6 | 1 | 1 | 0 | 1 | 1 | 0 | 0 | 0 | 0 | 1 | 1 |
| Whitall *et al.* [11] | 5 | 1 | 1 | 0 | 1 | 1 | 0 | 0 | 0 | 0 | 0 | 1 |
| Chouhan and Kumar [12] | 4 | 1 | 1 | 0 | 1 | 0 | 0 | 0 | 0 | 0 | 0 | 1 |
| Secoli *et al.* [13] | 4 | 1 | 1 | 0 | 1 | 0 | 0 | 0 | 0 | 0 | 0 | 1 |
| Thielman [14] | 6 | 1 | 1 | 1 | 1 | 1 | 0 | 0 | 0 | 0 | 0 | 1 |
| Johannsen *et al.* [15] | 6 | 1 | 1 | 0 | 1 | 1 | 0 | 0 | 0 | 0 | 1 | 1 |
| Stoykov *et al.* [16] | 4 | 1 | 1 | 0 | 1 | 0 | 0 | 0 | 0 | 0 | 0 | 1 |
| Richards *et al.* [17] | 4 | 1 | 1 | 0 | 1 | 0 | 0 | 0 | 0 | 0 | 0 | 1 |
| Jeong and Kim [18] | 5 | 1 | 1 | 0 | 1 | 0 | 0 | 0 | 0 | 0 | 1 | 1 |
| Waller and Whitall [19] | 6 | 1 | 1 | 0 | 1 | 1 | 0 | 0 | 0 | 0 | 1 | 1 |
| Luft *et al.* [20] | 4 | 1 | 1 | 0 | 1 | 0 | 0 | 0 | 0 | 0 | 0 | 1 |
| Thaut *et al.* [21] | 4 | 1 | 1 | 0 | 1 | 0 | 0 | 0 | 0 | 0 | 0 | 1 |
| Maulucci and Eckhouse [22] | 4 | 1 | 1 | 0 | 1 | 0 | 0 | 0 | 0 | 0 | 0 | 1 |
| Whitall *et al.* [23] | 6 | 1 | 1 | 0 | 1 | 1 | 0 | 0 | 0 | 0 | 1 | 1 |

Figure 1 Forest plot illustrating individual studies evaluating the effects of rhythmic auditory cueing on Fugl Meyer assessment scores on arm function amongst post stroke patients. Weighted effect sizes; Hedge’s g (boxes) and 95% C.I (whiskers) are presented, demonstrating repositioning errors for individual studies. The (Diamond) represents pooled effect sizes and 95% CI. A negative effect size indicated reduction in Fugl Meyer scores depicting poor arm functioning; a positive effect size indicated enhancement in Fugl Meyer scores depicting better arm functioning. (r-af: Real-time auditory feedback, low: Low performance group, high: High performance group, left CVA: Left sided cerebrovascular accident, right CVA: Right sided cerebrovascular accident)

Figure 2 Forest plot illustrating individual studies evaluating the effects of real-time auditory feedback (also for training 30 minutes or more per session, > 10 sessions) on Fugl Meyer assessment scores on arm function amongst post stroke patients. Weighted effect sizes; Hedge’s g (boxes) and 95% C.I (whiskers) are presented, demonstrating repositioning errors for individual studies. The (Diamond) represents pooled effect sizes and 95% CI. A negative effect size indicated reduction in Fugl Meyer scores depicting poor arm functioning; a positive effect size indicated enhancement in Fugl Meyer scores depicting better arm functioning. (r-af: Real-time auditory feedback, low: Low performance group, high: High performance group, left CVA: Left sided cerebrovascular accident, right CVA: Right sided cerebrovascular accident)

Figure 3 Forest plot illustrating individual studies evaluating the effects of rhythmic auditory cueing (30 minutes to 1-hour session, ≥ 3 sessions per week) on Fugl Meyer assessment scores on arm function amongst post stroke patients. Weighted effect sizes; Hedge’s g (boxes) and 95% C.I (whiskers) are presented, demonstrating repositioning errors for individual studies. The (Diamond) represents pooled effect sizes and 95% CI. A negative effect size indicated reduction in Fugl Meyer scores depicting poor arm functioning; a positive effect size indicated enhancement in Fugl Meyer scores depicting better arm functioning. (r-af: Real-time auditory feedback, low: Low performance group, high: High performance group, left CVA: Left sided cerebrovascular accident, right CVA: Right sided cerebrovascular accident)

Figure 4 Forest plot illustrating individual studies evaluating the effects of rhythmic auditory cueing on Wolf motor time assessment scores for arm function amongst post stroke patients. Weighted effect sizes; Hedge’s g (boxes) and 95% C.I (whiskers) are presented, demonstrating repositioning errors for individual studies. The (Diamond) represents pooled effect sizes and 95% CI. A negative effect size indicated reduction in Wolf motor scores depicting a better arm functioning; a positive effect size indicated enhancement in Wolf motor scores depicting poor arm functioning. (r-af: Real-time auditory feedback, low: Low performance group, high: High performance group, left CVA: Left sided cerebrovascular accident, right CVA: Right sided cerebrovascular accident)

Figure 5 Forest plot illustrating individual studies evaluating the effects of rhythmic auditory cueing (30 minutes to 1 hour, ≥ 3 sessions per week) on Wolf motor time assessment scores for arm function amongst post stroke patients. Weighted effect sizes; Hedge’s g (boxes) and 95% C.I (whiskers) are presented, demonstrating repositioning errors for individual studies. The (Diamond) represents pooled effect sizes and 95% CI. A negative effect size indicated reduction in Wolf motor scores depicting a better arm functioning; a positive effect size indicated enhancement in Wolf motor scores depicting poor arm functioning. (r-af: Real-time auditory feedback, low: Low performance group, high: High performance group, left CVA: Left sided cerebrovascular accident, right CVA: Right sided cerebrovascular accident)

Figure 6 Forest plot illustrating individual studies evaluating the effects of rhythmic auditory cueing and real-time auditory feedback on Action reach arm test on arm function amongst post stroke patients. Weighted effect sizes; Hedge’s g (boxes) and 95% C.I (whiskers) are presented, demonstrating repositioning errors for individual studies. The (Diamond) represents pooled effect sizes and 95% CI. A negative effect size indicated reduction in Action reach arm test depicting poor arm functioning; a positive effect size indicated enhancement in Action reach arm test depicting better arm functioning. (r-af: Real-time auditory feedback, low: Low performance group, high: High performance group, left CVA: Left sided cerebrovascular accident, right CVA: Right sided cerebrovascular accident)

Figure 7 Forest plot illustrating individual studies evaluating the effects of rhythmic auditory cueing and real-time auditory feedback on Nine-hole peg test among post stroke patients. Weighted effect sizes; Hedge’s g (boxes) and 95% C.I (whiskers) are presented, demonstrating repositioning errors for individual studies. The (Diamond) represents pooled effect sizes and 95% CI. A negative effect size indicated reduction in Nine-hole peg test depicting poor fine motor skills; a positive effect size indicated enhancement in Nine-hole peg test depicting better fine motor skills. (r-af: Real-time auditory feedback, low: Low performance group, high: High performance group, left CVA: Left sided cerebrovascular accident, right CVA: Right sided cerebrovascular accident)

Figure 8 Forest plot illustrating individual studies evaluating the effects of rhythmic auditory cueing and real-time auditory feedback on Stroke impact scale among post stroke patients. Weighted effect sizes; Hedge’s g (boxes) and 95% C.I (whiskers) are presented, demonstrating repositioning errors for individual studies. The (Diamond) represents pooled effect sizes and 95% CI. A negative effect size indicated reduction in Stroke impact scale depicting poor health status; a positive effect size indicated enhancement in Stroke impact scale depicting better health status. (r-af: Real-time auditory feedback, low: Low performance group, high: High performance group, left CVA: Left sided cerebrovascular accident, right CVA: Right sided cerebrovascular accident)

**References**

[1] D.-H. Bang, Effect of Modified Constraint-Induced Movement Therapy Combined with Auditory Feedback for Trunk Control on Upper Extremity in Subacute Stroke Patients with Moderate Impairment: Randomized Controlled Pilot Trial. Journal of Stroke and Cerebrovascular Diseases 25 (2016) 1606-1612.

[2] D.S. Scholz, S. Rhode, M. Großbach, J. Rollnik, and E. Altenmüller, Moving with music for stroke rehabilitation: a sonification feasibility study. Ann. N. Y. Acad. Sci. 1337 (2015) 69-76.

[3] M.P. Malcolm, C. Massie, and M. Thaut, Rhythmic auditory-motor entrainment improves hemiparetic arm kinematics during reaching movements: a pilot study. Top. Stroke. Rehabil. 16 (2009) 69-79.

[4] F. Speth, The role of sound in robot-assisted hand function training post-stroke, Humboldt-Universität zu Berlin, Kultur-, Sozial-und Bildungswissenschaftliche Fakultät, 2016.

[5] D.S. Scholz, S. Rohde, N. Nikmaram, H.-P. Brückner, M. Großbach, J.D. Rollnik, and E.O. Altenmüller, Sonification of Arm Movements in Stroke Rehabilitation – A Novel Approach in Neurologic Music Therapy. Front. Neurol. 7 (2016).

[6] A.L.E. van Delden, C.L.E. Peper, K.N. Nienhuys, N.I. Zijp, P.J. Beek, and G. Kwakkel, Unilateral versus bilateral upper limb training after stroke. Stroke (2013) STROKEAHA. 113.001969.

[7] G. Schmitz, D. Kroeger, and A.O. Effenberg, A mobile sonification system for stroke rehabilitation, Georgia Institute of Technology, 2014.

[8] J.-r. Kim, M.-y. Jung, E.-y. Yoo, J.-H. Park, S.-H. Kim, and J. Lee, Effects of rhythmic auditory stimulation during hemiplegic arm reaching in individuals with stroke: An exploratory study. Hong Kong Journal of Occupational Therapy 24 (2014) 64-71.

[9] E.M. Shahine, and T.S. Shafshak, The effect of repetitive bilateral arm training with rhythmic auditory cueing on motor performance and central motor changes in patients with chronic stroke. Egyptian Rheumatology and Rehabilitation 41 (2014) 8.

[10] D. Dispa, T. Lejeune, and J.-L. Thonnard, The effect of repetitive rhythmic precision grip task-oriented rehabilitation in chronic stroke patients: a pilot study. International journal of rehabilitation research 36 (2013) 81-87.

[11] J. Whitall, S.M. Waller, J.D. Sorkin, L.W. Forrester, R.F. Macko, D.F. Hanley, A.P. Goldberg, and A. Luft, Bilateral and unilateral arm training improve motor function through differing neuroplastic mechanisms a single-blinded randomized controlled trial. Neurorehabil. Neural. Repair. 25 (2011) 118-129.

[12] S. Chouhan, and S. Kumar, Comparing the effects of rhythmic auditory cueing and visual cueing in acute hemiparetic stroke. International Journal of Therapy & Rehabilitation 19 (2012).

[13] R. Secoli, M.-H. Milot, G. Rosati, and D.J. Reinkensmeyer, Effect of visual distraction and auditory feedback on patient effort during robot-assisted movement training after stroke. J. Neuroeng. Rehabil. 8 (2011) 21.

[14] G. Thielman, Rehabilitation of reaching poststroke: a randomized pilot investigation of tactile versus auditory feedback for trunk control. Journal of Neurologic Physical Therapy 34 (2010) 138-144.

[15] L. Johannsen, A.M. Wing, T. Pelton, K. Kitaka, D. Zietz, N. Brittle, P. van Vliet, J. Riddoch, C. Sackley, and R. McManus, Seated bilateral leg exercise effects on hemiparetic lower extremity function in chronic stroke. Neurorehabil. Neural. Repair. 24 (2010) 243-253.

[16] M.E. Stoykov, G.N. Lewis, and D.M. Corcos, Comparison of bilateral and unilateral training for upper extremity hemiparesis in stroke. Neurorehabil. Neural. Repair. 23 (2009) 945-953.

[17] L.G. Richards, C.R. Senesac, S.B. Davis, M.L. Woodbury, and S.E. Nadeau, Bilateral arm training with rhythmic auditory cueing in chronic stroke: not always efficacious. Neurorehabil. Neural. Repair. 22 (2008) 180-184.

[18] S. Jeong, and M.T. Kim, Effects of a theory-driven music and movement program for stroke survivors in a community setting. Applied Nursing Research 20 (2007) 125-131.

[19] S.M. Waller, and J. Whitall, Hand dominance and side of stroke affect rehabilitation in chronic stroke. Clinical rehabilitation 19 (2005) 544-551.

[20] A.R. Luft, S. McCombe-Waller, J. Whitall, L.W. Forrester, R. Macko, J.D. Sorkin, J.B. Schulz, A.P. Goldberg, and D.F. Hanley, Repetitive bilateral arm training and motor cortex activation in chronic stroke: a randomized controlled trial. Jama 292 (2004) 1853-1861.

[21] M.H. Thaut, G.P. Kenyon, C.P. Hurt, G.C. McIntosh, and V. Hoemberg, Kinematic optimization of spatiotemporal patterns in paretic arm training with stroke patients. Neuropsychologia 40 (2002) 1073-81.

[22] R.A. Maulucci, and R.H. Eckhouse, A real-time auditory feedback system for retraining gait. Conference proceedings : ... Annual International Conference of the IEEE Engineering in Medicine and Biology Society. IEEE Engineering in Medicine and Biology Society. Annual Conference 2011 (2011) 5199-202.

[23] J. Whitall, S.M. Waller, K.H. Silver, and R.F. Macko, Repetitive bilateral arm training with rhythmic auditory cueing improves motor function in chronic hemiparetic stroke. Stroke 31 (2000) 2390-2395.
